# Supplementary material for: Noise-tolerant single photon sensitive three-dimensional imager
Source: Nat Commun. 2020 Feb 17;11:921. doi: 10.1038/s41467-020-14591-8 (PMC7026101; doi:10.1038/s41467-020-14591-8)
Supplement: Supplementary file 1 — Supplementary Information [file 41467_2020_14591_MOESM1_ESM.pdf]

Supplementary Information

"Noise tolerant single photon sensitive three-dimensional imager"

Rehain et al.

Supplementary Information

Supplementary Note 1: Upconversion single photon detector characterization The fiber coupled upconversion single photon detector (USPD) consists of a temperature controlled PPLN waveguide, aspheric lenses and a series of optical filters as shown in Supplementary Figure 1. The length of the waveguide is 1.96 cm, and temperature is stabilized at 58.4± 0.1°C. With the aspheric lenses, input coupling efficiency from a single mode fiber is 34$\%$ and output coupling to a multimode fiber is 76$\%$. After the output coupling lens, a short pass filter (Cutoff wavelength: 950 nm, OD $>$ 5) is used to remove the residual pump pulses and three narrow bandpass filters (Center wavelength: 779.8 nm) were applied to provide out of band rejection of more than 140 dB. The series of filters are crucial to minimize the dark count of the upconversion module. To characterize the internal conversion efficiency and phase matching bandwidth of the PPLN, a continuous wave laser was coupled into the waveguide at transverse-electric polarization, and swept from 1555.8 to 1562.6 nm while measure the second harmonic power. The measured phase matching curve is centered at 1559.8 nm with the FWHM bandwidth of 0.73 nm and maximum normalized internal conversion efficiency of 121% W^{-1} cm^{-2}. The phase matching curve of PPLN waveguide is shown in Fig 2 where measurement data lie on top of a $sinc^2$ ideal phase matching profile, from which the inverse group velocity mismatch between upconversion and fundamental (signal and pump) wavelengths is calculated to be 282 $ps \cdot m^{-1}$ for our numerical simulation of mode selectivity.

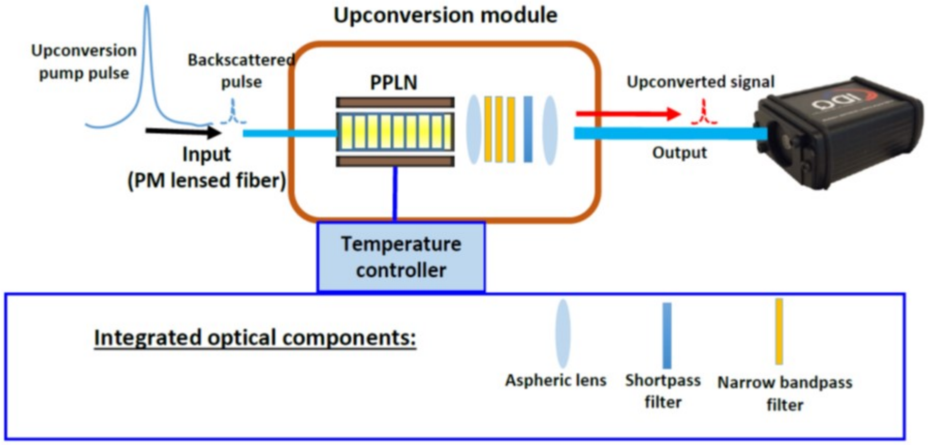

Supplementary Figure 1. The schematic diagram of fiber coupled upconversion module, integrating temperature controlled PPLN and a series of optical filters.

The pulse shape of the picoseconds probe and pump pulses carved out from the MLL with WDM filters are measured using a Frequency Resolved Optical Gating (FROG HR150) with 0.1 ps resolution. Both pulses are a in nearly Gaussian shape with FWHM 6.6 and 6.0 ps at 1554.1 nm and 1565.5 nm depicted in Fig. 4. Well understood amplitude and phase profile of pulses is crucial for the subsequent mode selective calculation, thus noise tolerant feature of our 3D imager. The total detection efficiency of our system is measured to be 3.6%, which includes the transmission loss of the filters, the free-space to fiber coupling loss, and the Si-APD's quantum efficiency (14 %) . The total detection efficiency can be improved to about 20 % by using a state-of-art Si-APD with quantum efficiency of 70 % at 780 nm.

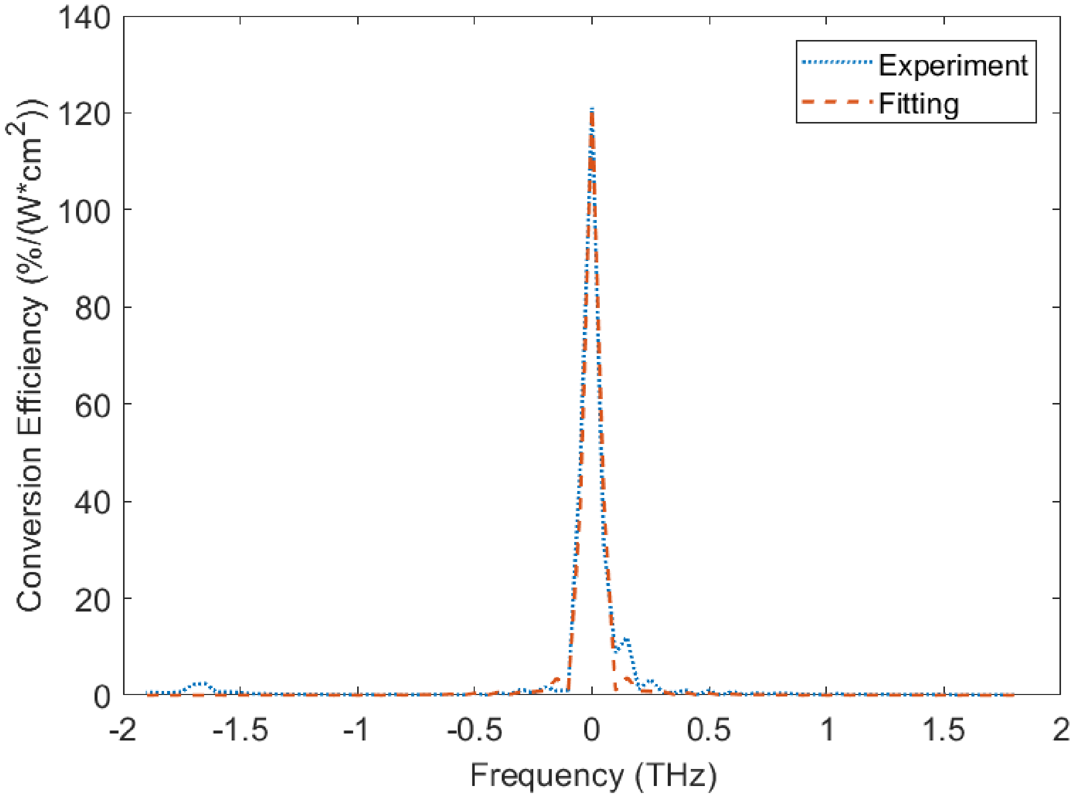

Supplementary Figure 2. Phase matching curve of the PPLN waveguide, plotted against the frequency offset at center wavelength of 1559.8 nm with a FWHM of about 80 GHz. The red line is the curve fitting, and the blue dashed line is the experimental result.

Supplementary Note 2: Dark noise of the imaging system The background noise of the imaging system is mainly attributed to the noise from the USPD. This consists of the intrinsic dark count rate of the Si-APD (50 Hz), and the noise of the upconversion module (200 Hz). In the upconversion module, noise photons can be generated in the SF band predominantly through two processes: (i) pump photons Raman scatterring into the signal band (centered at 1554.1 nm) then upconverting with the strong pump (centered at 1565.5 nm) via SFG, (ii) the Raman scattering of the SH light created by the pump. Operating the system at unity conversion efficiency with pump peak power of about 0.7 W (220 μW average power), the Raman noise photon count is about 200 Hz, as shown in Fig. 3, giving a total dark count rate of 250Hz per pixel. This corresponds to low noise probability of 5.0×10^{-6}/pulse/pixel due to the single detection mode of USPD [1].

Supplementary Note 3: Mode Selectivity of QPMS Noise rejection beyond the theoretical limit of linear-optical filtering is achieved via mode-selective quantum frequency conversion in a PPLN waveguide. The dynamics of nonlinear wave mixing are described by a set of coupled Heisenberg equations of motion [2,3]. For an undepleted pump pulse (shown in Fig 4(a)) propagating through the PPLN waveguide, input-output relations can be expressed using a Greens function. Then by modal decomposition, the normal modes and their eigenvalues can be calculated —where the eigenvalues represent the selection efficiency of each mode— see ref. 5 for detailed calculation procedures. A detailed profile and selection efficiency of each normal mode can be manipulated y engineering the phase matching properties of the PPLN waveguide

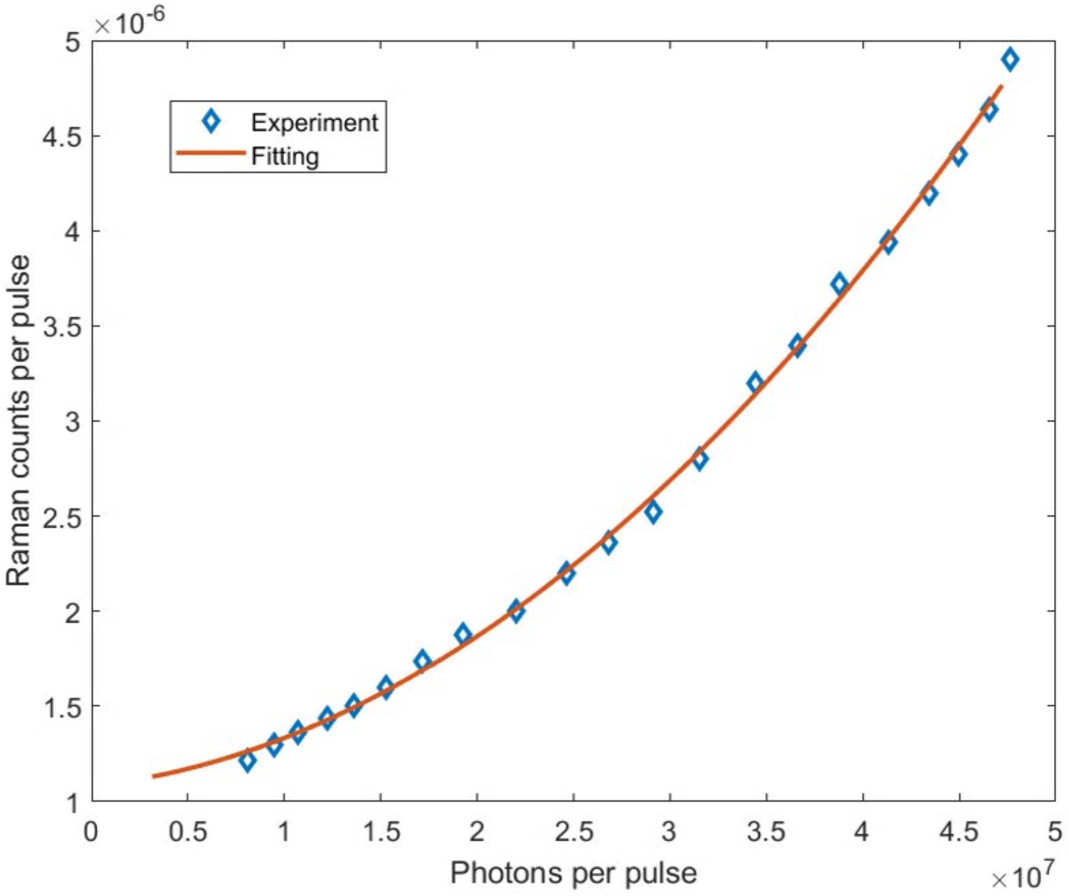

Supplementary Figure 3. The background count versus the input pump power. The diamond sign is the experiment background count, whose fitting curve (the red line) is a quadratic function.

and/or modulating the pump pulses [4,5]. In this work, we use a lithium-niobate waveguide that is periodically poled to phase match the SF generation between pump and probe pulses in telecom wavelengths. By using nearly transform limited pump and probe pulses, the current upconversion module effectively operates in the so-called single sideband velocity matched regime [2]. In this regime high mode-selectivity is enforced when the pumps spectrum exceeds the waveguides phase-matching bandwidth (Δ), or equivalently, the pumps temporal width is shorter than τ, where $\tau \sim 1/\Delta$ is the walk-off between the pump and SF waves in the waveguide. Here, considering the measured i) phase matching bandwidth, ii) intensity and phase profile for both pump and probe pulses, we numerically simulate the mode selectivity that can be achieved by this upconversion module. The mode selectivity is defined as the ratio of conversion efficiency of the spectral-temporal mode of probe pulse (η_1) compared to the conversion efficiency of all other modes occupying the identical spectral temporal domain. As shown in Fig 5(b), the mode selectivity defined as $S = \frac{\eta_1}{\sum_{n=2}^{10} \eta_n}$ of the dominating mode is calculated to be approximately 7.4 dB.

Supplementary Note 4: Longitudinal ranging accuracy calibration The longitudinal accuracy of our 3D imager was evaluated using a translation stage and a calibrated displacement indicator (Thorlabs DGM05) with resolution of 1 micron and accuracy of 2 micron. As shown in Fig 6(a), a flat metal block was stabilized on the linear translation stage, whose linear position was measured by using a displacement indicator. The laser beam was set parallel with the optical table's plane, illuminating a spot on the metal block, and the backscattered signal

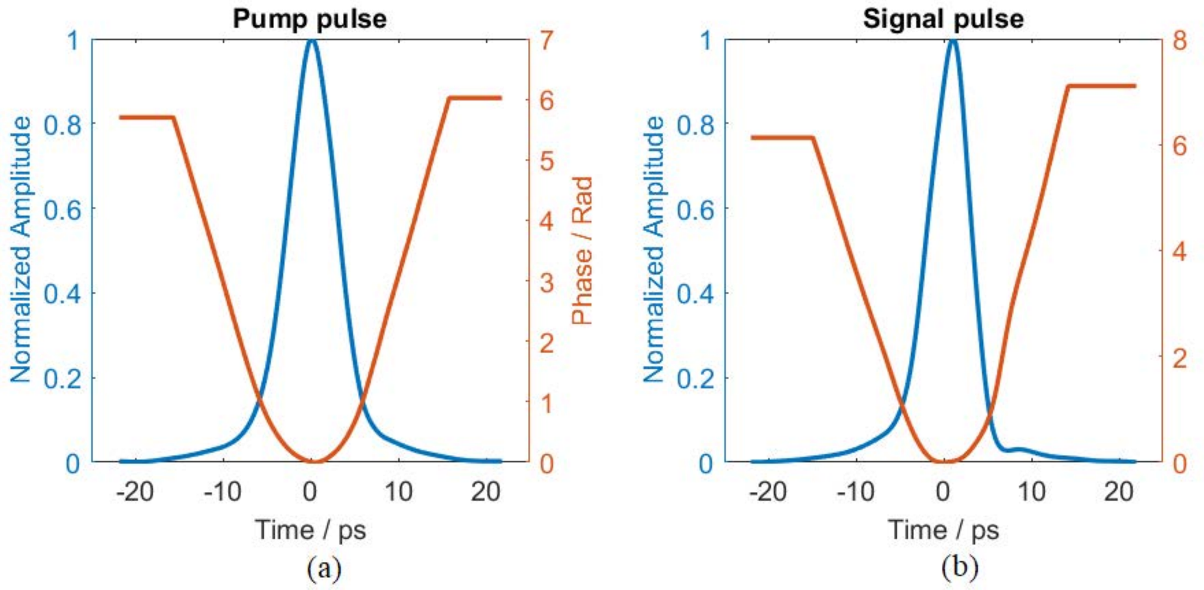

Supplementary Figure 4. Retrieved pulse shapes by the FROG. (a) and (b) show amplitude and phase profile of generated pulses at 1565.5 nm and 1554.1 nm, respectively. The pulse chirp is due to the flat top in the spectral profile of the commercial dense wavelength division multiplexers (DWDM-200 GHz) used to carve out probe and pump pulses from a mode-locked fiber laser.

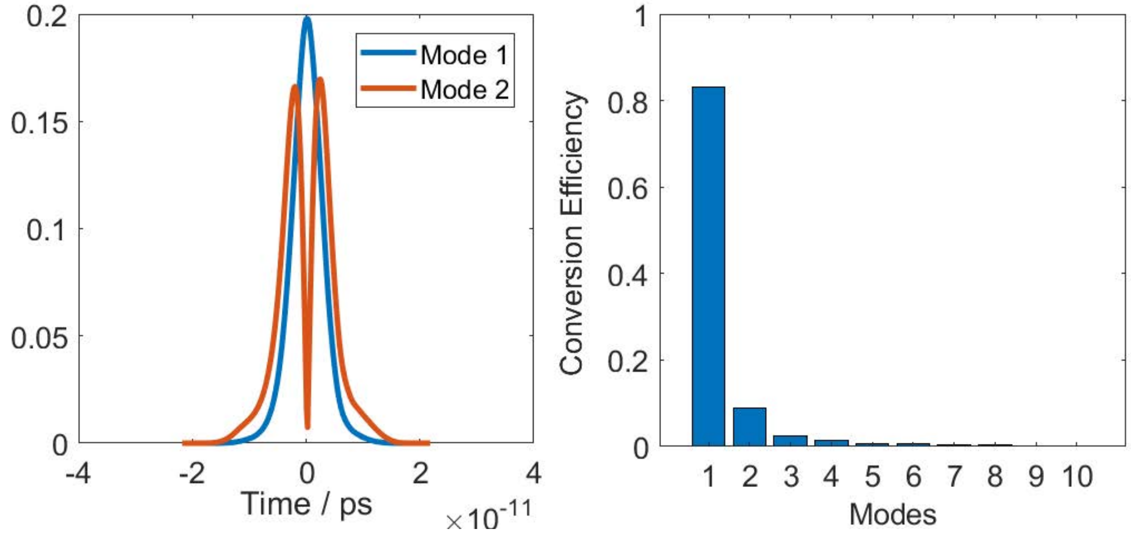

Supplementary Figure 5. (a) shows the first two modes of signal pulse. (b) Conversion efficiency between different modes for the pump. The calculated selectivity is 7.4 dB.

was detected using our 3D imager. The backscattered photons were attenuated to avoid saturating the detector. Using the translation stage, we carry out time resolved photon counting for depths incrementing in 0.15 mm steps. The longitudinal displacement measured by our system is compared with measurement obtained by the calibrated displacement indicator as shown in Fig 6(b). We define the longitudinal accuracy of our 3D imager as the deviation from the calibrated indicator measurement. The average longitudinal error is measured to be $\pm$ 0.09 mm.

Supplementary Note 5: Overcoming uncertainty caused by photon-number noise For a single maximum-likelihood value (MLV) estimate, the highest resolution this system can achieve is limited by the smallest step-size allowed by the optical delay line (ODL), which is

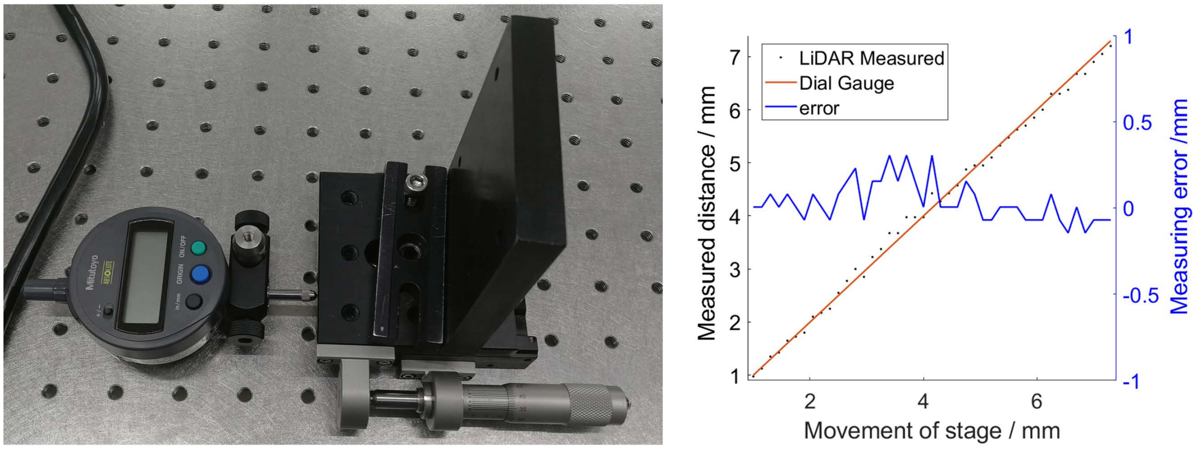

Supplementary Figure 6. (a) shows setup of the stage and the dial gauge. (b)The blue dots shows the measured points every 0.15 mm, and the red line shows the reading on the dial indicator The yellow line shows the longitudinal measurement error at each measurement point.

0.5ps. To reliably measure with this resolution there needs to be enough detections in the photon counting histogram to overcome photon-number noise and resolve the peak of the upconversion signal. To highlight the effects of photon-number noise Fig 7(b) and (c) show photon counting histograms for two levels of flux in the returning signal. As shown in Fig 7(a) we experimentally determined that 150 detections at the peak (corresponding to 0.006 detections per pulse over a 500μs integration time) are needed to accurately measure with the 0.5ps resolution set by the ODL. This was determined by calculating the root-mean-square (RMS) deviation in the MLV estimate versus the detections per pulse at the peak for repeated measurements on the same target, where a variable attenuator was used to reduce the flux of the returning signal.

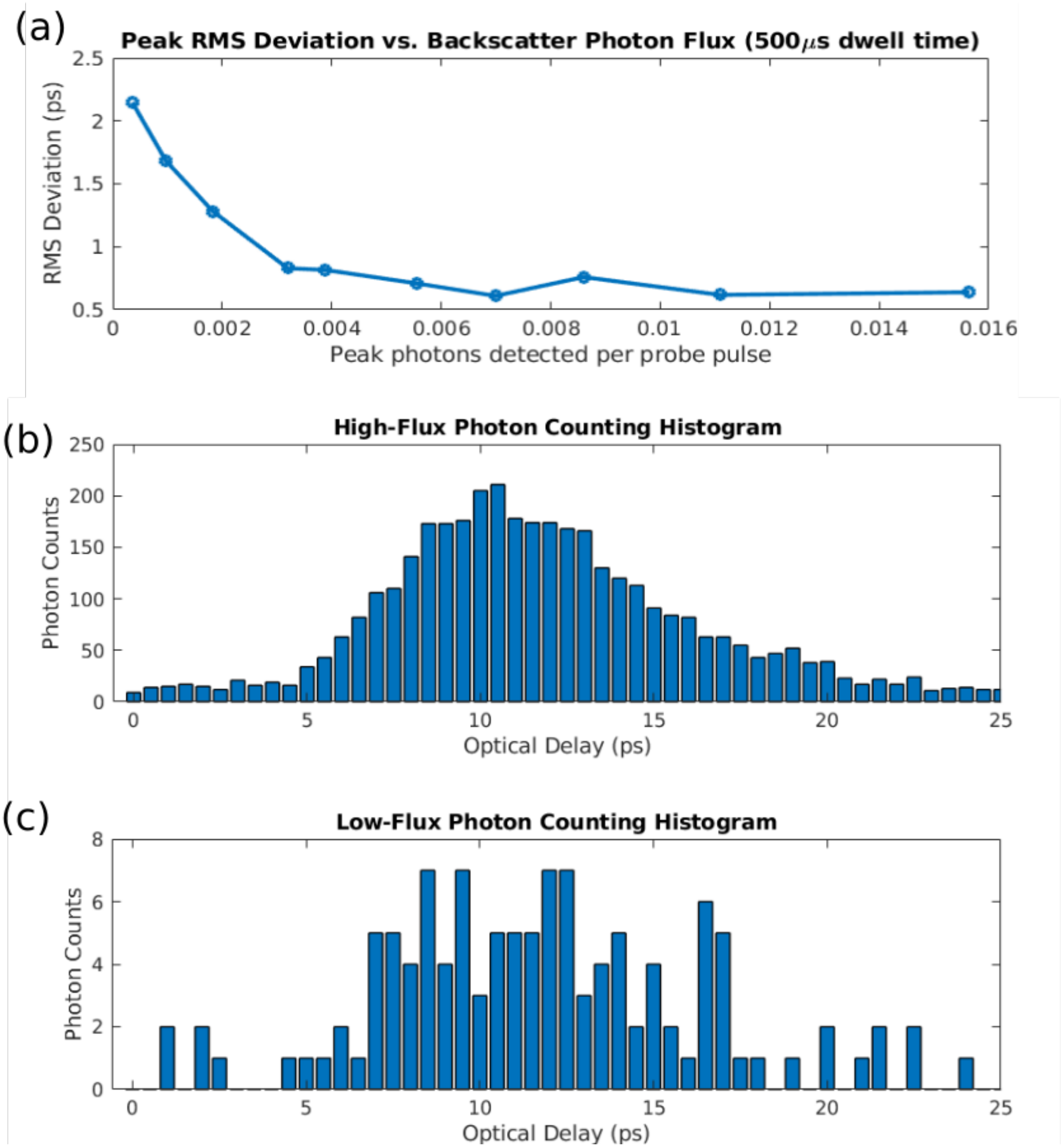

Supplementary Figure 7. (a)Plot of the RMS deviation in the temporal location of the peak versus the mean number of detections per pulse at the peak. (b)Shows a photon counting histogram used for the time-resolved measurement with a high-flux signal.(c)Shows a photon counting histogram used for the time-resolved measurement with a low-flux signal.

Supplementary Reference

1. Sua, Y. M., Fan, H., Shahverdi, A., Chen, J.-Y. & Huang, Y.-P. Direct generation and detection of quantum correlated photons with 3.2 um wavelength spacing. *Scientific Reports* **7**, 17494 (2017).

2. Reddy, D. V., Raymer, M. G., McKinstrie, C. J., Mejling, L. & Rottwitt, K. Temporal mode selectivity by frequency conversion in second-order nonlinear optical waveguides. *Opt. Express* **21**, 13840–13863 (2013).

3. Kowligy, A. S. *et al.* Quantum optical arbitrary waveform manipulation and measurement in real time. *Opt. Express* **22**, 27942–27957 (2014).

4. Huang, Y.-P., Altepeter, J. B. & Kumar, P. Heralding single photons without spectral factorability. *Phys. Rev. A* **82**, 043826 (2010).

5. Eckstein, A., Brecht, B. & Silberhorn, C. A quantum pulse gate based on spectrally engineered sum frequency generation. *Opt. Express* **19**, 13770–13778 (2011).
